# Correlation between the Lactate Dehydrogenase Levels with Laboratory Variables in the Clinical Severity of Sickle Cell Anemia in Congolese Patients

**DOI:** 10.1371/journal.pone.0123568

**Published:** 2015-05-06

**Authors:** Tite Minga Mikobi, Prosper Lukusa Tshilobo, Michel Ntetani Aloni, Georges Mvumbi Lelo, Pierre Zalagile Akilimali, Jean Jacques Muyembe-Tamfum, Valérie Race, Gert Matthijs, Jean Marie Mbuyi Mwamba

**Affiliations:** 1 Department des Sciences de Bases, Laboratory of Biochemistry and Molecular Biology, Faculty of Medicine, University of Kinshasa, Kinshasa, Democratic Republic of Congo; 2 Unit of Human Genetics, Department of Paediatrics, University Hospital of Kinshasa, School of Medicine, University of Kinshasa, Kinshasa, Democratic Republic of Congo; 3 Division of Hemato-oncology and Nephrology, Department of Paediatrics, University Hospital of Kinshasa, School of Medicine, University of Kinshasa, Kinshasa, Democratic Republic of Congo; 4 Division of Biostatistics and Epidemiology, School of Public Health, University of Kinshasa, Kinshasa, Democratic Republic of Congo; 5 Institut National de Recherche en Sciences Biomédicales, Kinshasa, Democratic Republic of Congo; 6 Center for Human Genetics, Katholieke Universiteit de Leuven, Leuven, Belgium; 7 Division of Hemato-Immuno-rheumatology, Department of Internal Medicine, University Hospital of Kinshasa, Faculty of Medicine, University of Kinshasa, Kinshasa, Democratic Republic of Congo; Université Claude Bernard Lyon 1, FRANCE

## Abstract

**Background:**

Sickle cell anemia is an inflammatory disease and is characterized by chronic hemolysis. We sought to evaluate the association of lactate dehydrogenase levels with specific clinical phenotypes and laboratory variables in patients with sickle cell anemia.

**Methods:**

The present cross-sectional study was conducted in Sickle Cell Centre of Yolo in Kinshasa, the Democratic Republic of Congo. Two hundred and eleven patients with Sickle Cell Anemia in steady state were recruited. Seventy-four participants with normal Hb (Hb-AA) were selected as a control group.

**Results:**

The average rates of hemoglobin, hematocrit, and red blood cells tended to be significantly lower in subjects with Hb-SS (p<0.001). The average rates of white blood cells, platelets, reticulocytes and serum LDH were significantly higher in subjects with Hb-SS (p<0.001). The average rates of Hb, HbF, hematocrit and red blood cells of Hb-SS patients with asymptomatic clinical phenotype were significantly higher than those of the two other phenotypes. However, the average rates of white blood cells, platelets, reticulocytes, and LDH of Hb-SS patients with the severe clinical phenotype are higher than those of two other clinical phenotypes. Significant correlations were observed between Hb and white blood cell in severe clinical phenotype (r3 = -0.37 *) between Hb and red blood cells in the three phenotypes (r1 = 0.69 * r2 * = 0.69, r3 = 0.83 *), and finally between Hb and reticulocytes in the asymptomatic clinical phenotype and severe clinical phenotype (r1 = -0.50 * r3 = 0.45 *). A significant increase in LDH was observed in patients with leg ulcer, cholelithiasis and aseptic necrosis of the femoral head.

**Conclusion:**

The increase in serum LDH is accompanied by changes in hematological parameters. In our midst, serum LDH may be considered as an indicator of the severity of the disease.

## Introduction

Sickle cell anemia (SCA) is one of the commonest genetic diseases worldwide [1]. SCA is an inflammatory disease and is characterized by chronic hemolysis [2–5].

The prevalence of sickle cell anemia is high in the Congolese population. The HBB*S allele frequency in neonates varies in the country, from 0.96% to 1.4% [6, 7]. However, there is no policy for early detection of the disease in the health care system and the diagnosis is delay in the majority of the patients [8]. It is well known that the early detection of SCA would provide the opportunity to implement adequate management to reduce the incidence of the acute crisis, transfusion rate and organ damage [9, 10].

An elevated serum of lactate dehydrogenase (LDH) was observed in the sickle cell patient population in steady state [11, 12]. The elevation of LDH was associated with hemolysis, pain crisis, pulmonary hypertension, leg ulcer, kidney damage and endothelial activation with elevated soluble vascular adhesion molecules [11–15]. However, SCA is clinically characterized clinically by a phenotypic polymorphism due to haplotype [16], genetics factors, fetal hemoglobin level [16, 17] and environmental factors [18]. All these factors may influence the severity of the disease and thus the level of LDH as shown in previous studies [13, 14, 19]. The identification of level of LDH may be considered as a marker of hemolysis and might be an important tool for the early detection of the severity of the disease in SCA individuals [13].

In the Democratic Republic of Congo (DRC), the Bantu haplotype is predominant and the Congolese SCA patients displayed low levels of fetal hemoglobin (HbF) and F-cells that contribute to the severity of SCA [20]. In addition, socio-economic conditions and the absence of a health care policy for the SCA patients exposed to severe forms [21, 22].

Despite this high prevalence and the risk severity of the disease, information about LDH in population suffering from SCA in DRC are unknown. The objective of this study was to investigate and determine the risk factors associated with clinical phenotypes among SCA individuals in steady state living in Kinshasa, the DRC.

## Materials and Methods

### Ethical considerations

All major participants provided written consent for study participation. Since some participants were minors, they provided oral assent and their legal guardians provided written consent for study participation. This consent procedure and the study were reviewed and approved by the National Ethical Committee of the Public Health School of the University of Kinshasa, Kinshasa, and the DRC (ESP/CE/027B/2011), in compliance with the principles of the Helsinki Declaration II. The aim and the procedures of the study were explained to the participants. The participants were informed that they could withdraw anytime without further obligation. None of the authors collected samples. Samples were collected and sent to the authors by Research unit of Sickle cell Centre of Yolo. Anonymity of the participants was guaranteed and no personal details were recorded.

### Study design and population

The present cross-sectional study was conducted in Sickle cell Centre of Yolo. These hospitals provide most of the non-private paediatrics beds in the DRC for sickle cell patients.

#### Study participants

The samples were collected from patients in steady state regularly followed up at the outpatient clinic of Sickle cell center of Yolo. All patients were free of pain for at one month and had not been hospitalized or transfused for at least 100 days before the study [23]. The starting number was randomly chosen from the first three in the section roll call. Every third patient was taken until the assigned number was reached.

We excluded subject with (i) initiated antibiotics treatment prior to seeking medical care; (ii) previous blood transfusion in the 3 months prior to the study (iii) under hydroxyurea (iv) under chronic transfusion program.

### Data collection procedure and blood analysis

Five ml of venous blood sample was drawn from each study participant into an EDTA tube, used to determine hematologic parameters. Hematologic parameters were performed using an automate Sysmex XS—1000 *i* (Lincolnshire, USA).

Five ml of venous blood sample was drawn from each study participant into an EDTA tube, used to determine haemoglobin electrophoresis. Sickle cell screening was performed using semi-automated electrophoresis technique with the Hydrasis II apparatus (SEBIA, France). The electrophoresis technique separates hemoglobin in acid and alkaline agarose gel. SCA was diagnosed in presence of production of Hb S with no Hb-A. The concentrations were measured by an integrated densitometer.

For LDH assay, the samples were collected in dry tubes. The serum LDH assay was performed with a spectrophotometer at 340 ηm with Thermo GENESYS 10S Bio apparatus (USA). The kit was provided by Cypress diagnostics (Landrop-Belgium). The reference values at 30°C were 160–320 U/L.

All analysis was performed at Institut National de Recherche Biomédicale (INRB) at Kinshasa, the DRC.

### Case definitions

We conceive a clinical phenotype score built up by recording the individual scores related to the most relevant medical history parameters. The following definitions were applied: asymptomatic clinical phenotype (ACP) (score ≤ 5), moderate clinical phenotype (MCP) (score between 6 and 15), and severe clinical phenotype (SCP) (score ≥ 16) (Table 1).

**Table 1:** Clinical criteria and severity score based on phenotype.

Clinical criteria	Variables	Score (points)
**Days of hospitalization/year**	≤ 1	0
	2–7	2
	≥ 8	5
**Severe vasoocclusive crisis/year**	0	0
	1–2	2
	≥ 3	5
**Blood transfusion/year**	0	0
	1–2	2
	≥ 3	5
**Hip disease**	Absent	0
	Present	5
**Leg ulcer**	Absent	0
	Present	5
**Hepatobiliary complications**	Absent	0
	Cholecystectomy	2
	Present	5
**Neurologic events**	Absent	0
	Present	5
**Renal disorders**	Absent	0
	Present	5
**BMI**	19–27	0
	< 19	2
**Total**	≤ 5: ACP* (1)	
	6–15: MCP** (2)	
	≥ 16: SCP*** (3)	

*ACP: Asymptomatic clinical phenotype;

**MCP: Moderate clinical phenotype;

***SCP: Severe clinical phenotype; BMI = Body Mass Index

Two hundred and eleven patients were suffering from SCA in steady state were recruited. All patients were homozygous for the β-globin S gene mutation (SS disease). Seventy-four participants with normal Hb (Hb-AA) were selected as a control group.

### Data management and analysis

Results were manually entered into a microcomputer and analyzed using the Excel Version 2002 (CDC) and they were exported on SPSS 17.0 for further analysis. Data are represented as means ± SD when the distribution was normal and median with range when the distribution was not normal. The analysis of Student's t-test was used for comparisons of means. ANOVA test were used to compare differences among categorical variables. Associations between variables and LDH was evaluated using chi-square and fisher exact test (for the cell with expected frequency less than 5 in two by two table more than 20%). Statistical significance level was set at p = 0.05.

## Results

### Age

The mean age of the patients with SCA was 21.2 (SD = 10.7) years while that of the control group was 29.75 (SD = 15.4) years (Table 2). In the SCA group, the mean age of the patients with ACP was 25.9 (SD = 10.0) years while that of the MCP subgroup was 20.5 (SD = 11.2) years and 18.9 (SD = 9.14) for patient with SCP (Table 3).

**Table 2:** Age and hematological parameters according hemoglobin electrophoresis status in the study population.

Variables	Group 1 (Hb-SS)	Group 2 (Hb-AA)	p*
**Age (years)**	21.2±10.7	29.8±15.4	0.001
**Hemoglobin (g/dL)**	7.7±1.7	12.5±1.8	0.001
**Hematocrit (%)**	23.5±5.0	39.3±4.8	0.001
**RBCs(x 10** ^**6**^ **/μL)**	3.0±0.8	4.9±0.6	0.001
**WBCs (x 10** ^**3**^ **/μL)**	12.6±6.2	5.2±1.2	0.001
**Reticulocytes (%)**	15.5±10.5	0.8±2.3	0.001
**Platelets (x 10** ^**3**^ **/μL)**	295.7±175.3	207.2±65.7	0.001
**MCV (fl)**	80±11	81±7	0.6
**MCHC (g/dL)**	33±2	32±1	0.001
**LDH (U/L)**	827±296	283±92	0.001

*Student test;

RBCs: Red Blood Cells; WBCs: White Blood Cells; MCV: Mean corpuscular volume; MCHC: Mean corpuscular hemoglobin concentration; LDH: Lactodehydrogenase

**Table 3:** Age and hematological parameters according clinical phenotypes of the study population.

variables	ACP n = 43	MCP n = 115	SCP n = 53	p* (*anova*)
**Age (years)**	25.9±10.0	20.5±11.2	18.9±9.1	0.003
**Hemoglobin (g/dl)**	9.1±1.7	7.5±1.3	6.8±1.6	0.001
**Hb F (%)**	15.7±7.4	4.6±2.9	0.0	0.001
**Hematocrit**	28±5	23±4	21±5	0.001
**RBCs(x 10** ^**6**^ **/μL)**	3.7±0.9	3.0±0.7	2.6±0.7	0.001
**WBCs(x10** ^**3**^ **/μL)**	8.9±4.1	13.0±6.3	15.0±6.0	0.001
**Reticulocytes (%)**	11.3±9.1	16.2±10.3	17.6±11.3	0.009
**Platelets (x10** ^**3**^ **/μL)**	249.1±104.4	288.4±157.4	349.1±236.9	0.02
**MCV (fl)**	79±12	79±10	84±12	0.01
**MCHC (g/dL)**	33±1	33±2	33±2	0.96
**LDH (U/I)**	705±299	840±294	897±274	0.01

*Anova test;

ACP: Asymptomatic clinic phenotype; MCP: Moderate clinic phenotype; SCP: Severe clinic phenotype; HbF: Fetal hemoglobin; RBCs: Red Blood Cells; WBCs: White Blood Cells; MCV: Mean corpuscular volume; MCHC: Mean corpuscular hemoglobin concentration; LDH: Lactodehydrogenase

The mean rate of HbF in SCA group was 6.35%.

### Gender

The sex-ratio male to female in the case group and control group was respectively 96/115 and 20/54 (Table 2). In the SCA group, the sex-ratio to female was respectively 11/32, 54/60 and 31/22 for patients with ACP, MCP and SCP (Table 3).

### Relationship between haematological variables in both groups

The average rates of Hb, hematocrit, and red blood cells were significantly lower in subjects with Hb-SS than in Hb-AA subjects (Table 2). The average rates of white blood cells, platelets, reticulocytes and serum LDH were significantly higher in subjects with Hb-SS than in Hb-AA subjects (Table 2).

### Relations between haematological variables in clinical phenotypes groups

The average rates of Hb, HbF, hematocrit and red blood cells of sickle cell patients with ACP clinical phenotype were significantly higher than those of the two other phenotypes MCP and SCP (Table 3). However, the average rates of white blood cells, platelets, reticulocytes, and LDH sickle cell patients with the SCP clinical phenotype SCP are higher than those of two other clinical phenotypes (Table 3).

Table 4 shows that significant correlations were observed between LDH and Hb in the ACP phenotype (r1 = -0.33 *) between the LDH and white blood cells in the ACP and MCP phenotypes (r1 = 0.51**, * r2 = 0.30), between LDH and reticulocytes in the ACP phenotype (r1 = 0.37 *).

**Table 4:** Correlation coefficients between LDH and haematological variables according clinical phenotype in the sickle cell study population.

variables	ACP (r_1_)	MCP (r_2_)	SCP (r_3_)
**Hemoglobin (g/dl)**	-0.33*	-0.11	-0.09
**WBCs (x 10** ^**3**^ **/μL)**	0.51**	0.30*	0.25
**Platelets (x 10** ^**3**^ **/μL)**	-0.24	0.18	-0.05
**Reticulocytes (%)**	0.37*	0.06	0.003

**: significant correlation at the 0.01 level;

*: significant correlation at the 0.05 level;

WBCs: White blood cells; LDH: Lactodehydrogenase

In relation with the Hb (Table 5), significant correlations were observed between Hb and white blood c in phenotype SCP (r3 = -0.37 *) between Hb and red blood cells in the three phenotypes (r1 = 0.69 * r2 * = 0.69, r3 = 0.83 *), and finally between Hb and reticulocytes in the ACP and SCP phenotypes (r1 = -0.50 * r3 = 0.45 *).

**Table 5:** Correlation coefficients between LDH and haematological variables according clinical phenotype in the sickle cell study population.

Variables	Correlation coefficient of Hb		
	ACP (r_1_)	MCP (r_2_)	SCP (r_3_)
**WBCs (x 10** ^**3**^ **/μL)**	-0.19	-0.15	-0.37*
**RBCs (x 10** ^**6**^ **/μL)**	0.69*	0.69*	0.83*
**Reticulocytes (%)**	-0.50*	-0.08	0.45*

*: Correlation significant at the 0.01 level;

WBCs: White blood cells; RBCs: Red blood cells

### Relationship between LDH and sickle cell complications

A significant increase in LDH was observed in patients with leg ulcer, cholelithiasis and aseptic necrosis of the femoral head in the SCP phenotype (Table 6).

**Table 6:** Relationship between clinical phenotypes, LDH and sickle cell complications.

Variables	ACP n = 43	MCP n = 115	SCP n = 53
**LDH (U/l)**	705±299	840±294	897±274
**VOC/year**	< 1	1–3	≥ 4
**Transfusion/year**	< 1	1–2	≥ 3
**Cholelithiasis (%)**	2	9.6	21.6
**Leg ulcers (%)**	1.6	10.1	23.5
**Femoral head necrosis**	3.6	15.9	36.0

## Discussion

LDH is considered a marker of hemolysis and an indicator of risk of morbidity and mortality in SCA [4, 11, 13, 15].

Our study showed that the values of Hb, Ht and red blood cells of sickle cell patients are lower than those of non-SCA subjects. The low rate of Hb in sickle cell patients is due to chronic hemolysis [4, 11, 15].

However, between sickle cell phenotypes we observed a difference in these values. In addition, our study showed a significant correlation between Hb and the number of red blood cells in the three phenotypes. The high rate of Hb and red blood cells in patients with ACP phenotype can be due to the presence of fetal hemoglobin [24, 25]. HbF is a genetic factor that modulates the sickle cell phenotype. The presence of fetal hemoglobin, has the effect of reducing the concentration of HbS in the erythrocytes and in the sickling process [26–28]. This dual action of HbF has the advantage of increasing the residual rate of Hb and to increase the survival time of red blood cells [26, 27,29, 30].

The mean of HbF in our study population was 6.35%. This rate is similar to that reported in previous African studies [20, 24, 25, 31–33]. However, the rate of HbF in our cohort was slightly low in comparison with arabo-indian haplotypes [34, 35].

In this cohort, the number of white blood cells of sickle cell patients was higher compared to non-SCD subjects. The factor responsible for leukocytosis in SCD steady state is not known. The analysis of the three phenotypes showed that there was a disparity of the white blood cells values number. The values were significantly higher in the ACP clinical phenotype. It has been shown that the leukocyte is an important factor that leads to the hyperviscosity and the phenomenon of endothelial adhesion [36–38]. The leukocytosis is associated with increased morbidity in sickle cell disease and can explain the difference in clinical expression of the disease between the three phenotypes as reported in previous studies [39].

Sickle cell patients have a higher reticulocytosis compared to non-SCA subjects. The hyper reticulocytosis is the result of the chronic peripheral hemolysis [40, 41].

However, between the different clinical phenotypes, our study has shown that patients with the ACP phenotype had lower reticulocytes values than the other two clinical phenotypes. This low rate of reticulocytes could be probably due to the presence of HbF leading to reduce hemolysis and to increase the survival time of erythrocytes F cells. From a pathological view, reticulocytes are associated with a risk of vaso-occlusive accidents because of the risk of adhesion to vascular endothelium [42]. In our study, hyper reticulocytosis was associated with higher morbidity in patients with SCP clinical phenotype. Our results confirm and extend previous observations [11, 43].

Our study confirmed that the SCA had a higher platelet count than non-SCA subjects. The platelet activation is an important factor in the pathogenesis of vaso-occlusive crises [44, 45]. During their activation, platelets secrete thrombospondin involved in vaso-occlusion [46]. Our study showed that between the different clinical phenotypes, the SCP clinical phenotype had high morbidity in relation with platelets activation.

Our study confirmed that SCA patients in steady state had a higher level of serum LDH than non-SCA subjects. This elevation of serum LDH is due to oxidative stress associated with chronic hemolysis [13].

Between the different clinical phenotypes, our study has shown that patients with the ACP phenotype had lower LDH values than the other two clinical phenotypes. The low level of LDH in patients with ACP phenotype may be due to a reduction of the oxidative stress associated with hemolysis [11, 47]. Our results showed that the decrease in hemolysis of patients with the ACP clinical phenotype is characterized biologically by an increase of Hb, a high number of red blood cells and a lower reticulocytosis. In addition, modulation of fetal hemoglobin in clinical severity was also demonstrated. In contrast, elevated serum LDH is associated with the SCP clinical phenotype as shown in Table 3, with the decrease of Hb rate, red blood cells and with an elevation of white blood cells, reticulocytes and platelets count. These hematological variables are associated with a high risk for increased mortality [11, 13].

In contrast, we observed a significant correlation between LDH and Hb in the ACP phenotype (r1 = -0.33 *) between LDH and white blood cells (r1 = 0.51 **) and between LDH and reticulocytes (r1 = 0.37 *). Several studies have shown that elevated levels of serum LDH was associated with hyperhemolysis, leg ulcers and acute chest syndrome [11, 13, 15].

## Conclusion

The first study conducted in Central Africa carried out on the Bantu population showed that the severity of the disease can vary within a haplotype from one individual to another depending on modulating factors. Our study confirmed that SCA patients in steady state have a high rate of Serum LDH. In addition, there is a correlation between the level of LDH and the severity of the disease. In SCA patients, serum LDH may be considered as an indicator of the severity of the disease in our midst.
